# Multimodal Adversarial Transfer Learning for Bearing Fault Diagnosis of Unmanned Mining Trucks in Realistic Noisy Environments

**DOI:** 10.3390/s26154890

**Published:** 2026-08-03

**Authors:** Haifeng Han, Rui Yang, Jianjian Yang, Chenyu Liu

**Affiliations:** 1 Inner Mongolia Research Institute, China University of Mining and Technology-Beijing, Ordos 017010, China; 18745606801@163.com (H.H.); yangjiannedved@163.com (J.Y.); 17377820925@163.com (C.L.); 2 School of Mechanical and Electrical Engineering, China University of Mining and Technology-Beijing, Beijing 100083, China

**Keywords:** unmanned mining truck, bearing fault diagnosis, data augmentation, adversarial transfer learning, multimodal fusion

## Abstract

Unmanned mining trucks operate in harsh environments such as those in open-pit mines, where online fault diagnosis of critical drivetrain bearings faces severe challenges including slow response, high precision requirements, and strong interference from realistic on-site noise. To address the insufficient generalization capability of existing diagnostic methods in real-world noisy scenarios, this paper proposes a multimodal adversarial transfer learning framework for bearing fault diagnosis in unmanned mining trucks. First, to bridge the domain shift gap between laboratory data and on-site truck data, an augmented multimodal dataset is constructed based on real-vehicle noise grafting. This approach fuses authentic background noise collected from the field with clean laboratory fault signals, thereby simulating graded on-site interference. Second, a deep feature extraction network integrating CNN, ViT, and CBAM attention mechanisms is designed. Building upon this backbone, an adversarial training scheme combined with a hierarchical adaptive fine-tuning strategy is introduced to formulate a domain-adversarial transfer learning model. This model is capable of extracting robust features that are both fault-discriminative and domain-invariant from multimodal signals (vibration and current). Experimental results on the constructed noise-augmented dataset demonstrate that the proposed method maintains high diagnostic accuracy in cross-domain scenarios with strong noise and limited samples, significantly outperforming conventional approaches. This study provides an effective technical pathway for real-time and highly reliable “edge-terminal” fault diagnosis of unmanned mining trucks operating in realistic noisy environments.

## 1. Introduction

With the advancement of intelligent mine construction, unmanned mining trucks have become core transportation equipment in open-pit mining operations, making their operational safety and reliability critically important [1]. Bearings, which serve as the fundamental supports for key rotating components such as wheel hubs and main reducers in mining trucks, are subjected to heavy loads, impact forces, and severe road-induced excitations over extended periods, resulting in frequent failures [2]. Once a failure occurs, a lack of timely early warning can readily lead to unplanned downtime or even major safety incidents [3]. Therefore, implementing online, rapid, and high-precision fault diagnosis for unmanned mining truck bearings constitutes a crucial element of the intelligent maintenance system [4]. However, the operating environment of mining trucks is complex, and onboard diagnostic terminals must contend with authentic and intense background noise interference, imposing stringent requirements on the generalization capability and robustness of diagnostic models [5].

Traditional bearing fault diagnosis methods largely rely on vibration signals acquired under ideal laboratory conditions and assume that the data follow an independent and identically distributed pattern [6]. In the actual operation of unmanned mining trucks, however, vibration signals contain not only fault-induced impulses but also complex broadband noise coupled from engine excitations, random road vibrations, and hydraulic system pulsations [7]. Under strong background noise, fault features are readily submerged in single-modality signals (e.g., using only vibration or current), leading to a sharp decline in diagnostic accuracy [8]. Moreover, existing studies are predominantly based on single operating conditions and struggle to accommodate the frequent variations in load and speed typical of mining truck operations [9]. Consequently, developing diagnostic methods capable of adapting to realistic noisy environments while effectively exploiting the complementarity of multi-source sensor information has become an urgent problem to be addressed [10].

In recent years, deep learning has driven the intelligent evolution of fault diagnosis [4]. On the one hand, multimodal fusion techniques provide a more comprehensive perspective on equipment health status by integrating heterogeneous sensor data such as vibration, current, and temperature [11].

In this study, the term “multimodal” refers to the fusion of heterogeneous information acquired from different sensing modalities. Specifically, vibration signals and motor current signals are regarded as two distinct sensing modalities because they originate from different sensing mechanisms and characterize the operating condition of the drivetrain from complementary physical perspectives. Vibration signals mainly reflect the mechanical dynamic behavior of the bearing and transmission system, whereas motor current signals capture the electrical response of the drive system under varying operating conditions. Their fusion enables the proposed framework to exploit complementary fault-related information, thereby improving the robustness and reliability of fault diagnosis under realistic noisy environments.

On the other hand, transfer learning—particularly domain-adversarial neural networks (DANNs) [12] and their variants—has been widely employed to address the domain shift problem, wherein the distribution of training data (source domain) differs from that of test data (target domain) [13]. Nevertheless, existing research still faces two critical challenges [14]: first, a pronounced domain shift exists between laboratory data and real-world scenarios, hindering direct deployment [15]; second, there is a lack of high-fidelity target domain data that accurately represent the on-site mining truck environment for model training and validation [16].

Deep feature learning-based methods have been widely applied to bearing fault identification for different types of bearings, including metallic, hybrid, and ceramic bearings. These methods can automatically extract discriminative features from vibration signals and achieve promising diagnostic performance. However, most existing approaches are developed under controlled laboratory conditions and may suffer from performance degradation when applied to realistic operating environments with strong noise interference and domain variations. Therefore, developing robust fault diagnosis methods with improved transferability remains a critical challenge for practical applications.

To address the above issues, this paper proposes a multimodal adversarial transfer learning framework for bearing fault diagnosis of unmanned mining trucks in realistic noisy environments. The main contributions are summarized as follows: Construction of a noise-grafted augmented dataset oriented toward real-vehicle scenarios: Moving away from the direct use of laboratory-conditioned data for training, this work proposes leveraging a background noise library collected from actual mining trucks. Through noise-grafting techniques, a multimodal augmented target domain closely resembling on-site conditions is constructed to reduce the gap between laboratory data and real-world operating environments, providing a data foundation for cross-domain model training and evaluation.

Design of a CNN–ViT–CBAM multimodal feature extraction network: By combining the local feature extraction capability of convolutional neural networks (CNNs) with the global dependency modeling strength of vision transformers (ViT), and by incorporating the convolutional block attention module (CBAM) to calibrate features, deep fusion and enhanced representation of vibration and current signals are achieved.

Development of a hierarchical adaptive fine-tuning adversarial transfer learning framework: An integrated adversarial transfer learning strategy is developed by combining domain alignment, adaptive weighting, and hierarchical fine-tuning mechanisms. These components jointly enhance domain-invariant feature learning and improve the generalization capability of the model under realistic noisy environments.

Although real-world fault-labeled data collected from operating mining trucks are extremely limited, the proposed study constructs a noise-grafted dataset that provides a controlled yet physically consistent approximation of realistic working conditions. By incorporating background noise acquired from actual mining trucks into laboratory fault signals while preserving cross-modal correlations, the constructed dataset enables systematic evaluation of model robustness under strong and complex noise interference.

To address the challenges of domain shift, multimodal fusion, and limited labeled data in realistic scenarios, this paper proposes a multimodal adversarial transfer learning framework tailored for bearing fault diagnosis of unmanned mining trucks. The proposed method is specifically designed for deployment in 300-ton-class mining trucks operating under harsh conditions characterized by strong vibration, dust interference, and dynamic load variations. By integrating physics-consistent data augmentation, hierarchical adaptive fine-tuning, and domain-invariant feature learning, the proposed framework effectively enhances the robustness and generalization capability of fault diagnosis models in realistic noisy environments.

## 2. Dataset

### 2.1. Laboratory Benchmark Dataset

**Figure 1:**
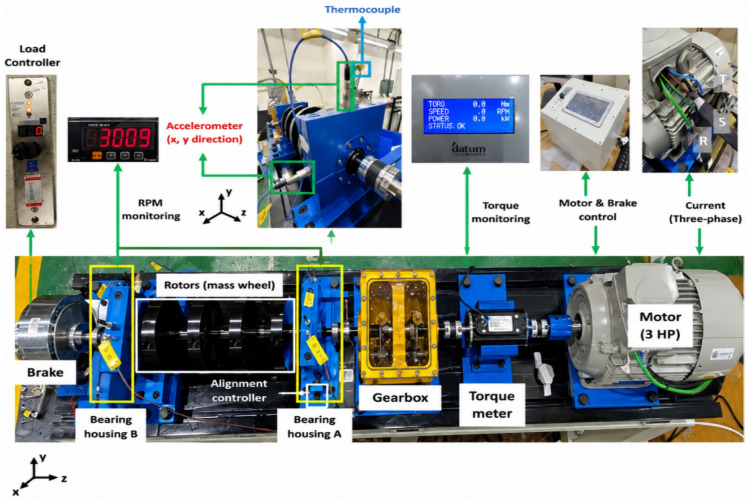
Korea Advanced Institute of Science and Technology (KAIST) [17].

The laboratory dataset used in this study originates from the publicly available bearing fault dataset released by the Noise and Vibration Control Laboratory, Department of Mechanical Engineering, Korea Advanced Institute of Science and Technology (KAIST), as shown in Figure 1 [17], as documented in the data paper by Wonho Jung et al. in Data in Brief. This dataset contains signals from two modalities, namely vibration and current, all of which are timestamped to ensure synchronous acquisition. It should be noted that the laboratory bearing components are not identical to those used in the mining truck. Instead, this dataset is utilized to extract general bearing fault vibration characteristics, as rolling-element bearing faults exhibit similar vibration generation mechanisms, including periodic impacts and friction-induced responses, across different mechanical systems Vibration signals were captured using four PCB 352C34 ceramic shear ICP accelerometers, and current signals were acquired using three Hioki CT6700 current transformers. The PCB 352C34 accelerometers have a sensitivity of approximately 100 mV/g, a measurement range of ±50 g, and a frequency response of approximately 5 Hz–10 kHz. The Hioki CT6700 current transformers (Hioki E.E. Corporation, Ueda, Japan) provide high-bandwidth current measurement with a frequency response of DC–50 MHz. Although the laboratory setup does not reproduce the exact structure of the mining truck, it provides representative bearing fault vibration patterns for vibration feature analysis and noise modeling. The dataset encompasses multiple bearing health conditions, including normal state, inner race fault, outer race fault, and rolling element fault. To meet the requirements of subsequent noise grafting, all signals in this dataset were uniformly subjected to anti-aliasing filtering and resampled to 12 kHz. The sensor specifications were obtained from the manufacturer datasheets.

### 2.2. On-Site Noise Acquisition and Analysis for Mining Trucks

**Figure 2:**
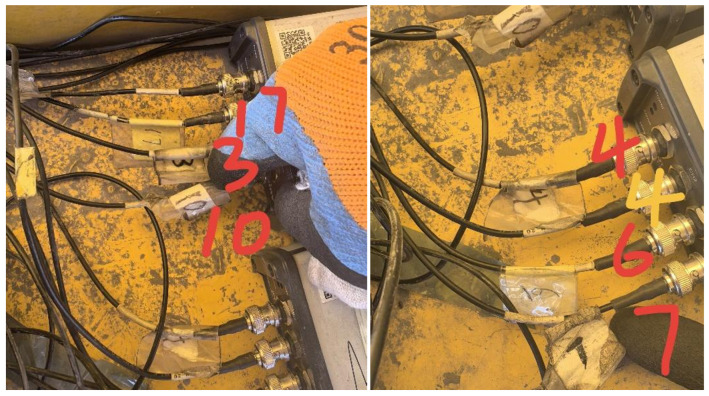
Eight-channel measurement point connection diagram.

**Figure 3:**
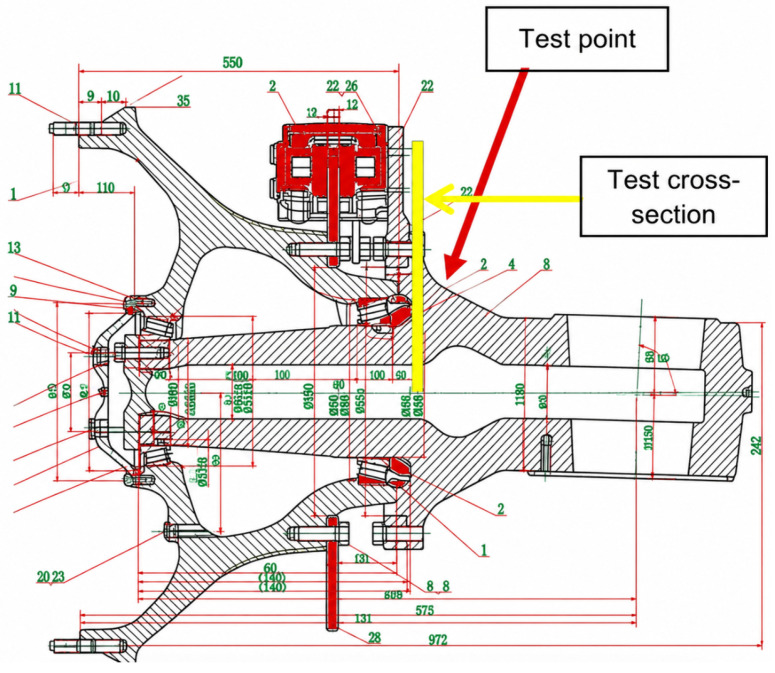
Test cross-section and test point layout.

**Figure 4:**
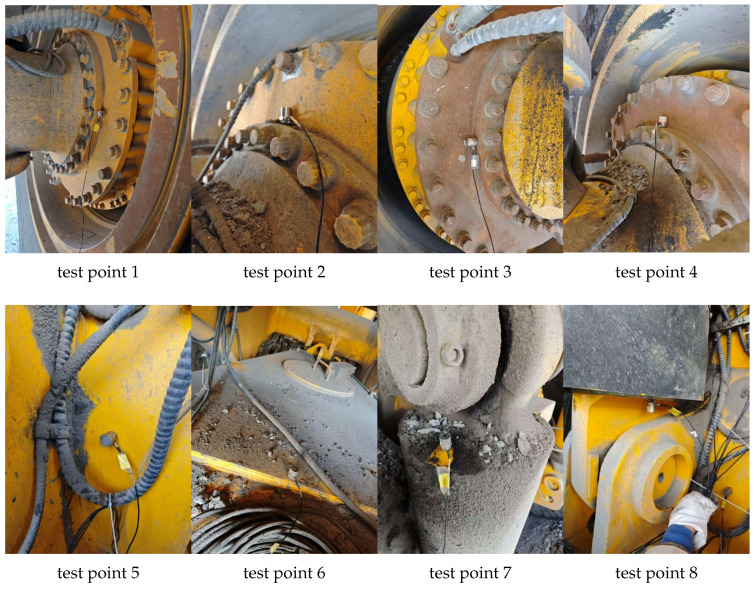
On-site measurement point layout.

To capture the background interference characteristics of an authentic noisy environment, an on-site noise acquisition test was conducted on a normally operating 300-ton-class mining dump truck in an open-pit mine. The test focused on the front wheel spindle bearing. In accordance with the GB/T 32333-2015 standard [18], eight vibration measurement points were deployed near the bearing, and one current measurement point was arranged at the wheel-side motor, as illustrated in Figure 2. A multi-channel synchronous acquisition system was employed to record vibration and current noise signals simultaneously. Among the eight vibration channels, four channels closest to the bearing load-bearing regions were selected for subsequent noise extraction, while the remaining channels were excluded due to stronger structural transmission and suspension-related disturbances. No PCA, averaging, or other mathematical transformation methods were applied. Instead, a physically informed channel selection strategy based on sensor locations and vibration transmission characteristics was adopted. The selected four vibration channels were directly matched with the four laboratory vibration channels to provide representative real-world background noise for noise grafting. The current noise signal was directly matched with the corresponding current modality. The test focused on the front wheel spindle bearing. The test cross-section and measurement point layout are illustrated in Figure 3 and the on-site arrangement of the vibration and current measurement points on the mining truck is shown in Figure 4.

It should be noted that the field-measured signals were collected from normally operating mining trucks and were used to characterize realistic background noise rather than provide fault-labeled field samples. Therefore, the target domain constructed in this study is a synthetic noisy target domain generated through noise grafting, rather than a real field fault domain.

The measurements were conducted during normal vehicle operation, and no fault alarms or abnormal operating behaviors were observed throughout the data acquisition process. The collected signals were carefully inspected, and segments containing obvious transient disturbances, abnormal impacts, or signal saturation were excluded to improve the reliability of the background noise library. Based on the measured vibration signals under different operating conditions, the vibration characteristics were analyzed in both the time and frequency domains. The results indicate that the dominant vibration components are mainly associated with periodic engine excitations, transient road-induced impacts, and broadband vibrations generated by the hydraulic system.

By applying zero-mean normalization and notch filtering, representative broadband background noise was extracted, thereby establishing a foundational noise library for the subsequent noise grafting procedure. During noise grafting, vibration and current signals were considered as two different sensing modalities. The extracted vibration noise was added only to the corresponding laboratory vibration signals, while the current noise was added to the laboratory current signals. This modality-specific noise injection strategy preserves the physical characteristics of each sensing modality and maintains the consistency of multimodal information. Therefore, the extracted noise mainly reflects the environmental interference and operational background noise naturally encountered during mining truck operation rather than fault-related responses. The core value of this real-vehicle dataset lies in providing a realistic background noise library rather than directly offering labeled fault samples. Therefore, the field-measured data are not considered as a real field fault domain. Instead, the target domain constructed in this study is a synthetic noisy target domain generated by incorporating field-measured background noise into laboratory fault signals through noise grafting. This strategy aims to reproduce realistic operating conditions while maintaining the fault characteristics contained in the laboratory dataset.

### 2.3. Noise Grafting Strategy and Augmented Dataset

To simulate the realistic noise environment of unmanned mining trucks, this study proposes a noise grafting strategy. First, the measured mining truck noise (original sampling rate of 10.24 kHz) is anti-aliasing filtered and resampled via interpolation to 12 kHz, consistent with the clean laboratory fault signals. Both are then truncated into samples of 4096 points each. To preserve the physical correlation between vibration and current modalities, the vibration noise and current noise used for grafting must be extracted from synchronously acquired segments of the same measurement period [19].

The signal-to-noise ratio (SNR) is defined according to Equation (1):

(1) $$\mathrm{SNR}=10\log_{10}\left(\frac{P_{\mathrm{signal}}}{P_{\mathrm{noise}}}\right)$$

The noisy signal $x_{\mathrm{noisy}}$ is generated by adding scaled noise to the clean signal $x_{\mathrm{clean}}$:

(2) $$x_{\mathrm{noisy}}=x_{\mathrm{clean}}+k\cdot n$$

**Figure 5:**
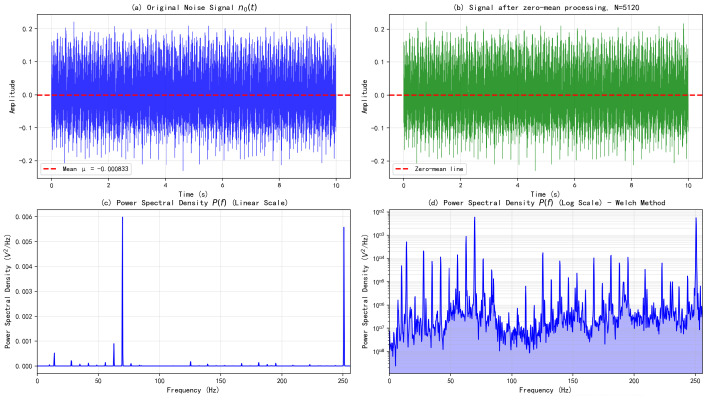
Vibration background noise processing and power spectral density.

**Figure 6:**
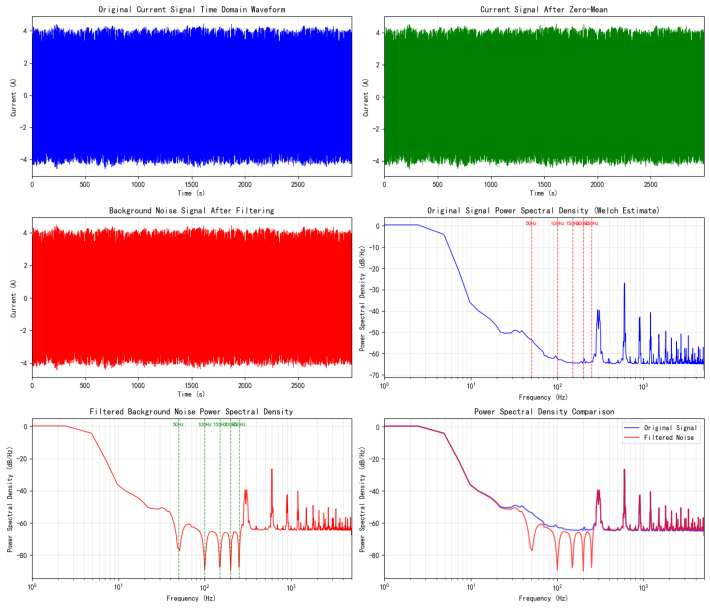
Current background noise processing and power spectrum comparison.

The vibration background noise processing results and the corresponding power spectral density analysis are shown in Figure 5. The logarithmic scale in the power spectral density plot uses scientific notation, where the values following 10 represent the corresponding exponents. The current background noise processing results and the corresponding power spectrum comparison are presented in Figure 6.

Let k be the scaling factor such that the resulting noisy signal satisfies the target SNR. Then, k is calculated as:

(3) $$k=\sqrt{\frac{P_{\mathrm{signal}}}{P_{\mathrm{noise}}}\cdot 10^{-\mathrm{SNR}/10}}$$

**Figure 7:**
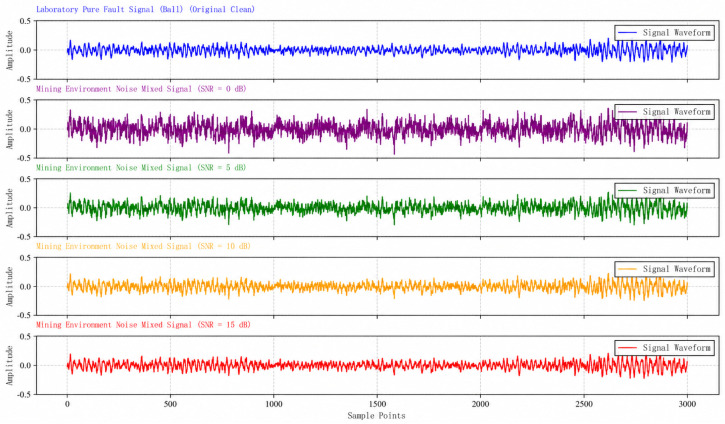
Time-domain waveform comparison before and after noise grafting.

Subsequently, the scaled noise segments are superimposed onto the vibration and current modalities of the laboratory fault signals according to Equations (1)–(3). To cover varying degrees of on-site interference, four target SNR levels are set: 15 dB, 10 dB, 5 dB, and 0 dB. Figure 7 presents a time-domain comparison of the signals before and after grafting. It is evident that the background noise is significantly intensified, with waveform fluctuations more closely resembling those of the actual measured mining truck data, while the fault-induced impulsive periodicity remains clearly discernible. Spectral analysis further confirms that the energy peaks at the fault characteristic frequencies are preserved, and the fault-related synchronicity between the vibration and current modalities remains intact, thereby validating the effectiveness of the proposed grafting strategy.

### 2.4. Quality Validation of the Augmented Dataset

To validate the effectiveness of the noise grafting strategy, a quantitative evaluation was conducted from three dimensions: data consistency, fault feature distinguishability, and noise suitability. Regarding data consistency, the scaling factor *k* was calculated according to Equations (2) and (3) to ensure that the resulting noisy signal $x_{\mathrm{noisy}}$ satisfies the preset SNR. Meanwhile, the vibration and current noise components were generated from synchronously acquired segments within the same measurement period, thereby preserving the physical correlation between different modalities. With respect to fault feature distinguishability, a fault feature energy ratio—defined as the ratio of the energy at the fault characteristic frequency to the total spectral energy—was introduced. The results show that even when the SNR drops to 0 dB, the energy peak at the fault characteristic frequency remains significantly higher than the background spectral level. Moreover, the impulsive periodicity induced by faults remains clearly identifiable in the time-domain signals. In terms of noise suitability, a baseline classification model (e.g., a CNN) was employed to evaluate diagnostic accuracy on the augmented dataset under different SNR levels. As shown in Table 1, when the SNR decreases from 15 dB to 0 dB, the classification accuracy of the baseline model gradually declines from 96.2% to 82.5%. This trend indicates that the proposed augmented dataset not only preserves the intrinsic discriminative features of the original faults but also realistically simulates mining truck operating environments ranging from low noise to extremely strong noise conditions. Therefore, it provides a graded benchmark for evaluating the robustness of the proposed method.

## 3. Fault Diagnosis Model Based on Adversarial Transfer Learning

### 3.1. Overall Framework of the Model

**Figure 8:**
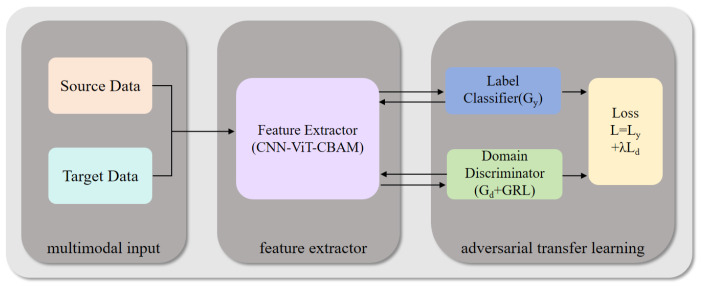
Architecture of the proposed adversarial transfer learning framework.

The proposed adversarial transfer learning fault diagnosis model is illustrated in Figure 8, which consists of three core modules: a feature extractor, a label classifier, and a domain discriminator. The feature extractor employs the main body of the CNN–ViT–CBAM architecture (with the classification layer removed) to map the multimodal input into a high-dimensional feature space. The label classifier is responsible for decoding fault category information from the deep features and computes the classification loss exclusively on the source domain. The domain discriminator is connected to the feature extractor via a gradient reversal layer (GRL), thereby establishing an adversarial learning mechanism. Specifically, the domain discriminator endeavors to accurately distinguish the origin of the features (source domain or target domain), whereas the feature extractor seeks to maximize the domain classification loss. This adversarial interplay compels the extracted features to simultaneously possess fault discriminability (by minimizing the classification loss) and domain invariance (by confusing the domain discriminator), ultimately enabling knowledge transfer from laboratory-augmented data to real-vehicle operating conditions.

### 3.2. Feature Extractor: CNN–ViT–CBAM

**Figure 9:**
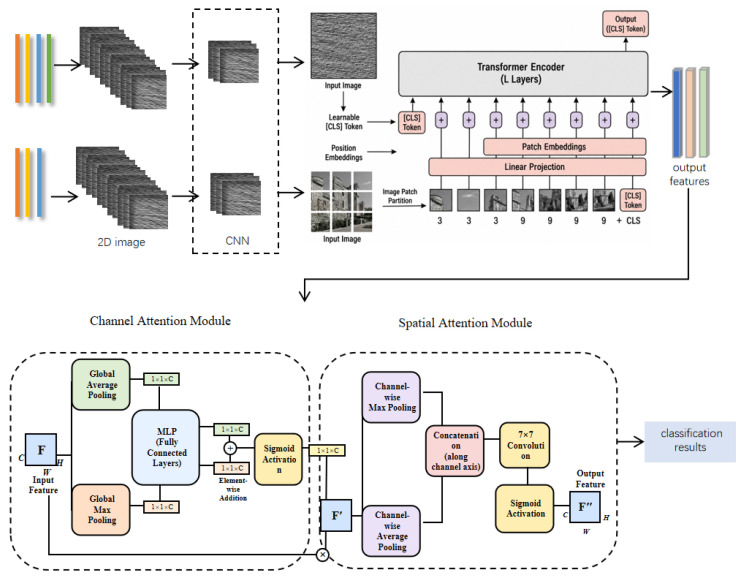
Overall structure of the CNN–ViT–CBAM model.

The overall architecture of the proposed CNN–ViT–CBAM model is depicted in Figure 9. The model comprises four serially connected modules: a data preprocessing and dual-stream CNN feature extraction module, a dual-stream ViT global feature enhancement module, a multimodal feature fusion and CBAM calibration module, and a classification module.

First, the raw vibration and current signals are preprocessed. The vibration signal consists of four channels, corresponding to the horizontal and vertical directions of the two bearing housings. The current signal comprises three channels, i.e., the three-phase currents. Each sample contains 256 data points, which are normalized to the range of 0 to 255 using min–max normalization. After preprocessing, the vibration signal is reshaped into a 16×16×4 grayscale image, and the current signal is reshaped into a 16×16×3 grayscale image.

Two parallel CNN branches are then employed to process the vibration and current images separately. The two branches share an identical architecture but have independent parameters, as detailed in Table 2. Each branch consists of three convolutional layers with 3×3 kernels. The first layer maps the input channels to 64 feature maps; the second layer maintains 64 channels; and the third layer increases the channel dimension to 128, which is subsequently reduced back to 64 via a 1×1 convolution. The spatial resolution of the feature maps remains 16×16 throughout the CNN module. The resulting feature maps are then fed into the ViT module. Each feature map is divided into 4×4 patches, resulting in a total of 16 patches. Each patch is flattened and linearly projected into a 128-dimensional embedding vector, followed by the addition of learnable positional encoding. The sequence is processed through four Transformer encoder layers, each equipped with 8-head multi-head self-attention. Finally, the output corresponding to the class token is extracted as the global feature representation of each modality, denoted as $F_{\mathrm{vib}}$ and $F_{\mathrm{cur}}$, both with a dimension of 128.

The two global feature vectors are concatenated to obtain a 256-dimensional fused representation. To accommodate the CBAM module, the fused vector is first mapped through a fully connected layer and reshaped into a three-dimensional feature map $M\in R^{4\times 4\times 64}$. The CBAM consists of a channel attention module and a spatial attention module, which are applied sequentially. In the channel attention module, both global average pooling and global max pooling are applied to *M* to generate two channel descriptors. These descriptors are passed through a shared multi-layer perceptron (MLP), summed element-wise, and activated by a sigmoid function to produce channel attention weights. The weights are then multiplied with *M* to perform channel-wise feature recalibration. In the spatial attention module, average pooling and max pooling are first performed along the channel dimension of the channel-refined feature map. The resulting two feature maps are concatenated and fed into a 7×7 convolution layer followed by a sigmoid activation function to generate spatial attention weights. These weights are multiplied with the input feature map to obtain the refined feature representation $M^{\prime \prime }$.

Finally, global average pooling is applied to $M^{\prime \prime }$ to obtain the final feature vector, which is fed into a fully connected layer with a softmax activation function to output the predicted probabilities of fault categories. The entire network is optimized using the cross-entropy loss function. The proposed CNN–ViT–CBAM model serves as the backbone network for the subsequent adversarial transfer learning framework, enabling effective extraction of both local and global discriminative features from multimodal signals.

### 3.3. Adversarial Training Mechanism and Joint Loss Function Design

(1) Principle of the Gradient Reversal Layer

The gradient reversal layer (GRL) acts as an identity transformation during forward propagation, while during backpropagation it multiplies the gradient by a negative scalar, thereby achieving an adversarial effect. Its mathematical definition is as follows:

(4) GRL(f)=f

(5) $$\frac{d(GRL(f))}{df}=-\lambda I$$

where λ is a trade-off parameter that controls the degree of domain confusion.

The implementation of the GRL ensures that the gradient of the domain discriminator loss is reversed when backpropagated to the feature extractor. That is, the feature extractor updates its parameters in the direction that increases the domain classification loss, thereby forcing the extracted features to be indistinguishable with respect to their domain of origin.

(2) Joint Loss Function

The overall loss function comprises a source domain classification loss $L_{y}$ and a domain discrimination loss $L_{d}$.

Source domain classification loss $L_{y}$: standard cross-entropy loss, defined as:

(6) $$L_{y}=-\frac{1}{n_{s}}\sum_{i=1}^{N_{s}}\sum_{k=1}^{K}y_{i}^{k}\log\left({\hat{y}}_{i}^{k}\right)$$

Domain discrimination loss $L_{d}$: binary cross-entropy loss that measures the domain discriminator’s ability to distinguish source and target domain features:

(7) $$L_{d}=-\frac{1}{n_{s}+n_{t}}\sum_{i=1}^{n_{s}+n_{t}}\left[d_{i}\log\left({\hat{d}}_{i}\right)+\left(1-d_{i}\right)\log\left(1-{\hat{d}}_{i}\right)\right]$$

The overall adversarial objective is:

(8) $$L_{\mathrm{total}}=L_{y}-\lambda L_{d}$$

where λ is a trade-off parameter. Minimizing $L_{\mathrm{total}}$ requires minimizing $L_{y}$ while maximizing $L_{d}$, thereby encouraging the feature extractor to learn fault-discriminative yet domain-invariant representations.

(3) Dynamic Trade-off Strategy

To balance classification accuracy in the early stage of training with domain alignment effectiveness in the later stage, a dynamically adjusted trade-off parameter λ is employed:

(9) $$\lambda_{p}=\frac{2}{1+e^{-\gamma p}}-1$$

where *p* represents the current training progress (ranging from 0 to 1), and γ controls the growth rate (set to γ=10 in this study).

This strategy ensures that λ is relatively small at the beginning of training, allowing the model to focus on learning class-discriminative features. As training progresses, λ gradually increases, thereby strengthening the domain alignment effect. Such dynamic adjustment circumvents the issue of degraded classification performance that may arise from enforcing domain alignment prematurely.

### 3.4. Hierarchical Adaptive Fine-Tuning Strategy

The core innovation of this section lies in adapting the CNN–ViT–CBAM model, proposed in Section 3.2, to the adversarial transfer learning framework. This adaptation enables the model to maintain its powerful feature extraction capability while learning domain-invariant features through adversarial training.

(1) Pre-trained Model Inheritance Strategy

To fully leverage the prior knowledge learned from the source domain data, a two-stage training strategy is adopted:

Stage 1 (Pre-training): The CNN–ViT–CBAM model is trained on the constructed augmented multimodal dataset to obtain a feature extractor with parameters $\theta_{f0}$ and a classifier with parameters $\theta_{y0}$ that possess strong fault-discriminative capabilities.

Stage 2 (Adversarial Transfer): The pre-trained parameters are loaded as initialization. A domain discriminator is introduced, and the model is fine-tuned through adversarial training to adapt to the target domain distribution.

(2) Hierarchical Fine-Tuning Strategy

Features extracted by different network layers exhibit varying degrees of generalization capability: shallow layers tend to extract generic features (e.g., edges, textures), whereas deeper layers extract task-specific features. Based on this principle, a hierarchical fine-tuning strategy is designed, as detailed in Table 3. The advantage of hierarchical fine-tuning is twofold: it preserves the general knowledge already acquired by the pre-trained model while endowing the model with sufficient flexibility to adapt to the target domain distribution, thereby effectively mitigating overfitting in small-sample scenarios.

The advantage of hierarchical fine-tuning is twofold: it preserves the general knowledge already acquired by the pre-trained model while endowing the model with sufficient flexibility to adapt to the target domain distribution, thereby effectively mitigating overfitting in small-sample scenarios.

### 3.5. Domain-Adaptive Enhancement via Multi-Scale Feature Fusion

To enhance the domain invariance of the fused features, a domain-adaptive enhancement mechanism is introduced at the feature fusion stage. By dynamically balancing local details and global semantic information, this mechanism improves the model’s robustness to cross-domain distribution discrepancies. The refined feature fusion formulation is defined as follows:

(10) $$f_{\mathrm{fusion}}=\alpha \cdot f_{\mathrm{cnn}}+\left(1-\alpha \right)\cdot f_{\mathrm{vit}}+\beta \cdot \left(f_{\mathrm{cnn}}⊙f_{\mathrm{vit}}\right)$$

where $f_{\mathrm{cnn}}\in R^{D}$ denotes the local texture features output by the CNN branch (reinforced by CBAM), and $f_{\mathrm{vit}}\in R^{D}$ denotes the global contextual features output by the ViT branch; both are of dimension 128.

The fusion process comprises two key components. First, a weighted linear combination dynamically adjusts the proportion of local and global features via a learnable scalar parameter α (initialized to 0.5 and optimized through backpropagation). This enables the model to adaptively select the dominant feature representation based on sample characteristics. Second, a nonlinear interaction term $\beta \cdot (f_{\mathrm{cnn}}⊙f_{\mathrm{vit}})$ is included, where ⊙ represents element-wise multiplication and β is a trainable interaction weight. This interaction term is designed to capture higher-order correlations between local and global features, reinforcing their synergistic activations at corresponding spatial positions and thereby extracting richer discriminative information.

The dynamic adjustment mechanism for β is coupled with the adversarial training process. Under the influence of the gradient reversal layer in the domain discriminator, the update of β is governed not only by the classification loss but also by the objective of domain confusion. When the distribution discrepancy between the source and target domains is large, the adversarial training encourages β to increase, thereby strengthening the contribution of the interaction term and steering the fused features toward the common representations shared by local and global views. Conversely, when the domain discrepancy is small, β decreases correspondingly, preventing feature redundancy caused by excessive coupling. This adaptive mechanism enables the fused features to suppress domain-related noise components while preserving fault-sensitive information, thereby enhancing the domain invariance of the feature representation. Ultimately, the fused feature vector $f_{\mathrm{fusion}}$ serves as the shared input to both the label classifier and the domain discriminator, further reinforcing the alignment capability of the adversarial transfer learning framework.

To underscore the innovative aspects of this work, a comparison between the proposed model and the standard DANN is presented in Table 4.

It should be noted that the adversarial alignment strategy, adaptive weighting mechanism, and interaction term are jointly designed as components of the transfer learning framework rather than independent feature extraction modules. Therefore, their effects are evaluated through the overall performance improvement of the complete model.

## 4. Experimental Validation and Results Analysis

### 4.1. Experimental Setup

The dataset is partitioned into a source domain *S* and a target domain *T*. The source domain adopts the KAIST laboratory dataset described in Section 2.1, which comprises four bearing health states across 36 distinct operating conditions (three load levels × three rotational speeds), with 500 samples per condition. The target domain is constructed following the noise grafting procedure detailed in Section 2.3, wherein real-vehicle background noise is superimposed onto the source signals at a signal-to-noise ratio (SNR) of 10 dB.

To simulate a practical small-sample scenario, the target domain training set contains only 10 labeled samples per class, while the test set comprises 200 samples per class. The source domain is randomly split into training and validation sets at a ratio of 7:3. Labeled target samples are used exclusively for computing the classification loss $L_{y}$, whereas the unlabeled target samples participate jointly with source samples in the adversarial domain alignment process.

Considering the limited availability of labeled fault data in real mining truck scenarios, a small-sample target-domain setting was adopted to evaluate the generalization capability of the proposed method under realistic conditions. Specifically, the target domain training set contains only 10 labeled samples per class, while the test set comprises 200 samples per class. The source domain is randomly split into training and validation sets at a ratio of 7:3. Labeled target samples are used exclusively for computing the classification loss $L_{y}$, whereas unlabeled target samples participate jointly with source samples in the adversarial domain alignment process. All experiments were independently repeated five times, and the average results were reported to reduce random variations and ensure the reliability of the evaluation.

Three cross-domain evaluation scenarios are established: cross-device, and small-sample. All experiments are independently repeated five times, and the average results are reported.

Implementation details: The experiments are conducted using the PyTorch 1.13 framework on a workstation equipped with an Intel i7-12700H CPU and an NVIDIA RTX 3060 GPU. The Adam optimizer is employed with an initial learning rate of 0.001 and a batch size of 32. Training proceeds for a maximum of 100 epochs with early stopping (patience = 10). The trade-off parameter λ is dynamically increased according to Equation (9) during adversarial training. In the hierarchical fine-tuning phase, the shallow CNN layers and the CBAM module are frozen, while the remaining modules are updated with a reduced learning rate of $1\times 10^{-4}$. During hierarchical fine-tuning, the CBAM module is frozen while the domain adaptation-related layers are updated. This strategy is based on the consideration that CBAM mainly performs feature recalibration by assigning adaptive weights to informative channels and spatial regions. Such feature enhancement characteristics are relatively stable across different domains. Freezing CBAM reduces unnecessary parameter updates and helps maintain stable feature recalibration during transfer learning.

### 4.2. Comparison of Cross-Domain Diagnostic Performance

Table 5 summarizes the fault diagnosis accuracy of the four methods under different scenarios. As can be observed from the table, the CNN–ViT–CBAM model without transfer learning exhibits the poorest performance across all scenarios, with accuracy falling below 50% in the cross-device scenario, directly demonstrating the severe constraint imposed by domain shift on model generalization. Both DANN and CDAN effectively mitigate domain shift through adversarial training, achieving accuracy improvements of 15 to 25 percentage points over the non-transfer approach. CDAN, owing to the introduction of conditional discrimination, slightly outperforms DANN in complex cross-domain settings.

The proposed method attains an accuracy of 89.5% on the real-vehicle noise dataset, representing a 5.7 percentage point improvement over CDAN. This result validates the effectiveness of the hierarchical adaptive fine-tuning strategy in addressing structural domain shift. Notably, in the small-sample scenario, the model without transfer learning still maintains a baseline accuracy of 68.8% due to its pre-trained knowledge. However, the proposed method further elevates the accuracy to 92.1% through adversarial transfer, underscoring that the domain adaptation mechanism is more critical than relying solely on pre-training when labeled target data are scarce.

**Figure 10:**
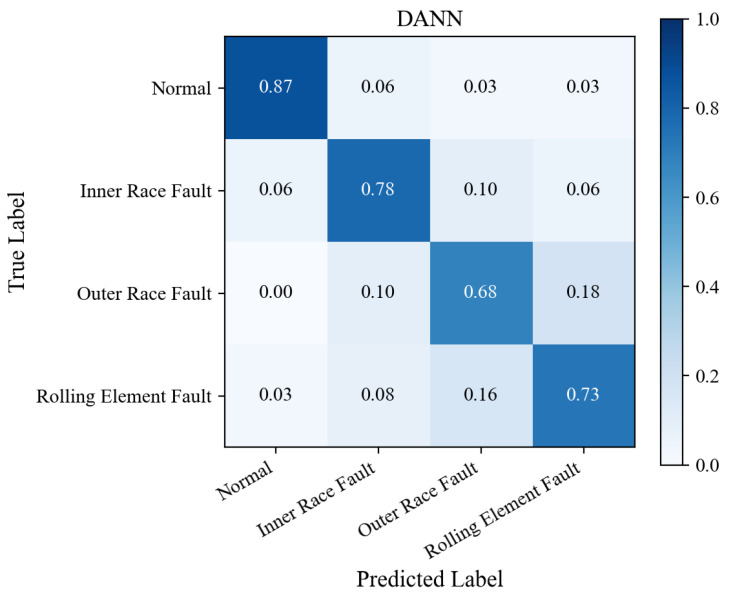
Confusion matrix of DANN (cross-device scenario).

**Figure 11:**
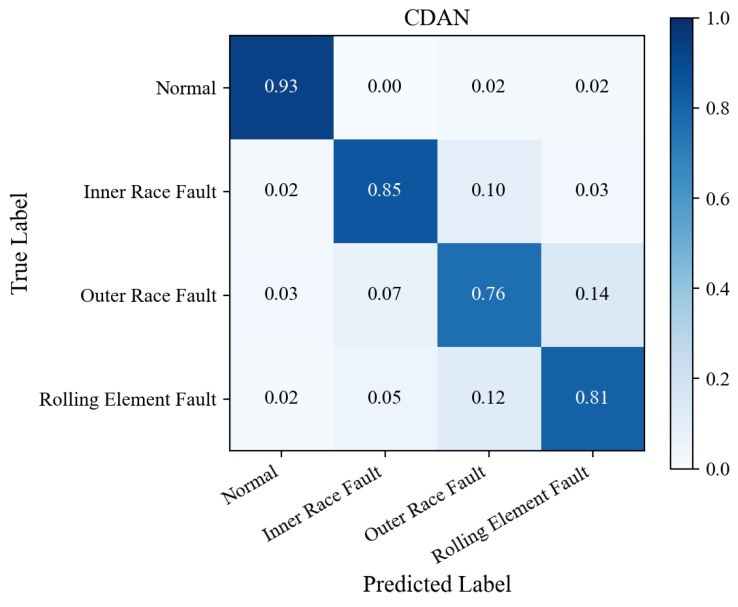
Confusion matrix of CDAN (cross-device scenario).

**Figure 12:**
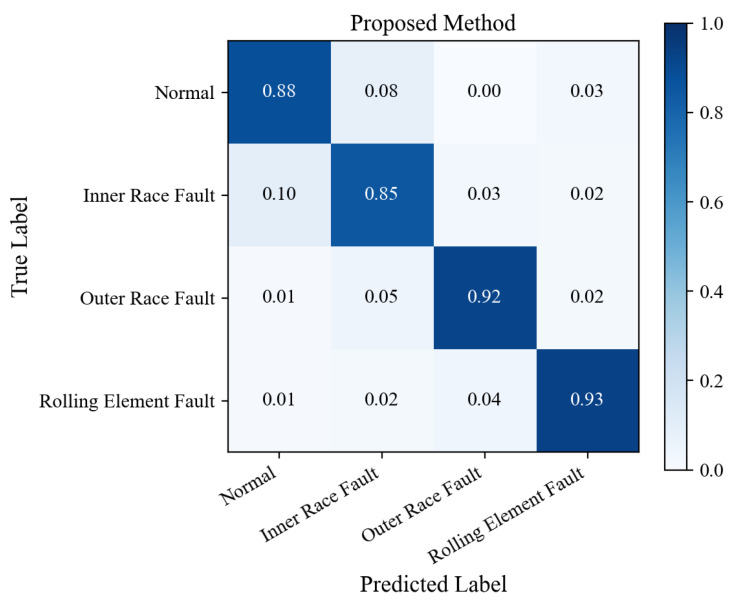
Confusion matrix of the proposed method (cross-device scenario).

Figure 10, Figure 11 and Figure 12 present the confusion matrices of DANN, CDAN, and the proposed method under the cross-device scenario, respectively. DANN (Figure 10) exhibits notable confusion between outer race and rolling element faults, with misclassification rates of 18% and 16%. CDAN (Figure 11) reduces these rates to 14% and 12% via conditional discrimination, though some confusion persists. In contrast, the proposed method (Figure 12) yields a highly diagonalized confusion matrix, with outer-race-to-rolling-element and rolling-element-to-outer-race misclassification rates dropping to merely 4% and 3%, respectively. Furthermore, inner race fault recognition improves from 80% (DANN) to 95% (proposed), confirming the model’s balanced discriminative capability across all fault categories.

### 4.3. Ablation Study

To systematically evaluate the contributions of the major architectural components in the proposed model, including multimodal fusion, ViT-based global feature extraction, and the CBAM attention mechanism, an ablation study is conducted. These experiments mainly focus on the effectiveness of the feature representation architecture, while the adversarial transfer learning components are designed as an integrated optimization strategy within the complete framework.

The following clear conclusions can be drawn from Table 6, where the bold values indicate the best performance among all evaluated models:

Effectiveness of multimodal fusion: Model B fuses vibration and current features via simple concatenation, and its accuracy improves by 3.6 percentage points compared with the best single modality (vibration). This confirms the complementary nature of information from heterogeneous sensor signals.

Contribution of ViT-based global feature enhancement: Model C further introduces the ViT branch on top of the fused features, raising the accuracy to 95.3%. This indicates that the global dependencies captured by the Transformer architecture facilitate the differentiation of easily confusable fault modes (e.g., rolling element faults versus outer race faults).

High cost-effectiveness of the CBAM module: Model D (the proposed model) achieves the highest accuracy of 96.8% after incorporating CBAM. Notably, CBAM adds merely 0.5 M parameters (a relative increase of approximately 10%) while delivering a performance gain of 1.5 percentage points, which is comparable to the gain brought by the ViT module (which adds 3.5 M parameters). This convincingly demonstrates that calibrating feature maps through channel and spatial attention constitutes an efficient and powerful optimization approach.

In summary, the ablation study verifies that the major feature representation components in the proposed CNN–ViT–CBAM architecture contribute positively to the final performance. The results demonstrate the effectiveness of multimodal fusion and attention-based feature enhancement. The adversarial transfer learning strategy and related optimization mechanisms are integrated into the overall framework to improve domain adaptation capability under realistic noisy environments.

### 4.4. Computational Efficiency Analysis

The parameter counts, floating-point operations (FLOPs), and per-sample inference times (on GPU) of the compared models are summarized in Table 7. As shown in the table, although the proposed model has a higher parameter count (5.2 M) and computational cost (1.8 G FLOPs) than the single-modality CNN, its inference time is merely 7.3 ms, which is well below the 50 ms threshold typically required for real-time diagnosis of unmanned mining trucks. Compared with the CNN–ViT model, the inclusion of CBAM adds only 0.5 M parameters and 0.3 G FLOPs while delivering a nearly 2% improvement in accuracy, exhibiting outstanding cost-effectiveness. Considering both performance and efficiency, the proposed model is well-suited for deployment on edge computing devices in unmanned mining trucks, possessing the potential for lightweight implementation and a solid foundation for real-time operation.

## 5. Conclusions

Focusing on the urgent demand for online bearing fault diagnosis of unmanned mining trucks operating under realistic noisy conditions, this paper proposes an intelligent diagnostic framework that integrates real-vehicle noise augmentation with multimodal adversarial transfer learning. The main conclusions are summarized as follows:

A real-vehicle noise grafting strategy is proposed to construct a multimodal noisy dataset that better reflects practical operating environments of mining trucks. Experimental results demonstrate that the generated noisy samples preserve clear fault-related impulse characteristics under an SNR of 10 dB, providing an effective basis for evaluating cross-domain fault diagnosis methods.

A CNN–ViT–CBAM-based feature extraction network combined with a hierarchical adaptive fine-tuning adversarial transfer framework is developed to effectively exploit complementary information from vibration and current signals. The proposed method achieves superior diagnostic performance under cross-noise, cross-device, and small-sample scenarios, demonstrating its capability to extract domain-invariant fault features under complex operating conditions.

The proposed framework achieves a good balance between diagnostic accuracy and computational efficiency. With only 5.2 M parameters and an inference time of 7.3 ms per sample, the model exhibits strong potential for real-time implementation on edge computing platforms for unmanned mining trucks. Moreover, the ablation results verify that the CBAM module improves diagnostic performance with limited additional computational cost.

Despite the promising performance, this study has some limitations. Due to the difficulty of collecting sufficient fault-labeled data from operating mining trucks, the target domain used in this work is constructed by combining field-measured background noise with laboratory fault signals rather than using fully real-world fault data. Future research will focus on collecting more field fault samples, further validating the proposed framework under practical operating conditions, and exploring lightweight deployment strategies such as model compression and quantization. In addition, the generalization capability of the proposed method will be investigated for other critical mining truck components, including gearboxes and wheel-side motors.

Overall, the proposed method provides an effective solution for reducing the gap between laboratory-based fault diagnosis models and practical intelligent maintenance applications in unmanned mining trucks, offering a promising pathway toward reliable online condition monitoring in intelligent mining systems.

Future work will investigate the integration of additional sensing modalities, such as acoustic and stray flux signals, by developing modality-specific feature extraction and adaptive fusion strategies to further improve the robustness of the proposed framework under complex operating conditions.

## Figures and Tables

**Table 1:** Performance comparison of noise-grafted datasets under different signal-to-noise ratios.

Dataset	SNR (dB)	Fault Feature Energy Ratio	Baseline CNN Accuracy (%)
Original data	∞	0.85	98.5
Grafted data	15	0.72	96.2
Grafted data	10	0.58	93.8
Grafted data	5	0.41	89.1
Grafted data	0	0.26	82.5

**Table 2:** Detailed network structure parameters of the CNN–ViT–CBAM model [20].

Module	Layer Name	Output Size	Parameter Description
Input Layer	Input (Vibration)	16×16×4	Vibration grayscale image
	Input (Current)	16×16×3	Current grayscale image
CNN Module	Conv2d-1	16×16×64	3×3 conv, 64 kernels, padding = 1
	Conv2d-2	16×16×64	3×3 conv, 64 kernels, padding = 1
	Conv2d-3	16×16×64	3×3 conv, 64 kernels, padding = 1
ViT Module	Patch Embedding	16×128	Patch size 4×4, projection dim 128
	Position Encoding	16×128	Learnable positional encoding
	Transformer Encoder × 4	16×128	Multi-head attention (8 heads)
	Class Token Output	128	Class token used as global feature
CBAM	Concatenate	256	Concatenation of vibration and current features
Classification Module	FC Mapping Layer	4×4×64	Fully connected layer; outputs 1024-dim then reshapes
	Channel Attention	4×4×64	Channel attention (MLP reduction ratio 16)
	Spatial Attention	4×4×64	Spatial attention (7×7 conv)
	Global Average Pooling	64	Spatial average pooling
	FC Classification Layer	4	Fully connected layer, Softmax activation

**Table 3:** Fine-tuning strategies and theoretical basis for individual branch modules in adversarial training.

Network Module	Layer Range	Update Strategy	Theoretical Basis
CNN Branch	First 2 convolutional layers	Frozen	Extract generic local features shared across domains
	Last 2 convolutional layers	Fine-tuned	Require adaptation to target domain-specific noise patterns
CBAM Module	Channel and spatial attention	Frozen	Attention weights provide relatively stable feature recalibration across domains
ViT Branch	First 4 Transformer layers	Fine-tuned	Global dependencies require cross-domain adaptation
	Last 2 Transformer layers	Fine-tuned	High-level semantic features demand focused adaptation
Feature Fusion Layer	Adaptive weighted fusion	Fine-tuned	Adjusts the contribution weights of CNN and ViT branches
Classifier($G_{y}$)	Fully connected layers	Retrained	Classification boundaries may shift with domain change

**Table 4:** Comparative analysis between the proposed model and standard DANN.

Comparison Dimension	Standard DANN	Proposed Model	Innovative Advantage
Feature Extractor	Simple CNN	CNN–ViT + CBAM attention	Simultaneously captures local details and global dependencies.
Input Modality	Single signal	Multimodal (vibration + current)	Complementary information enables more robust fault diagnosis.
Pre-training Strategy	Random initialization/simple pre-training	Inherits optimized model	Fully leverages knowledge learned from the source domain.
Fine-tuning Strategy	Uniform fine-tuning of all layers	Hierarchical adaptive fine-tuning	Mitigates overfitting under small-sample conditions.
Feature Fusion	None or simple concatenation	Adaptive weighting + interaction enhancement	Enhances feature representation and domain invariance.

**Table 5:** Comparison of accuracy of different algorithms under cross-domain scenarios.

Cross-Domain Scenario	CNN–ViT–CBAM (*w*/*o* Transfer)	DANN	CDAN	Proposed Method
Cross-device	46.8	76.5	83.8	89.5
Small-sample	68.8	72.8	75.4	92.1
Average Accuracy	55.3	73.6	76.0	90.8

**Table 6:** Comparison of ablation experiment results.

Model	Modality	CNN	ViT	CBAM	Accuracy (%)	Parameters (M)	Performance Gain
Baseline 1	Vibration only	✓			90.2	0.8	–
Baseline 2	Current only	✓			86.5	0.8	–
Model B	Vibration + Current	✓			93.8	1.2	+3.6/+7.3 (vs. Baseline 1/2)
Model C	Vibration + Current	✓	✓		95.3	4.7	+1.5 (vs. Model B)
Model D (Ours)	Vibration + Current	✓	✓	✓	**96.8**	5.2	+1.5 (vs. Model C)

**Table 7:** Comparison of computational efficiency among different algorithms.

Model	Parameters (M)	FLOPs (G)	Inference Time (ms)
Single-modal CNN (Vibration)	0.8	0.12	2.1
Feature Concatenation Fusion	1.2	0.18	2.8
CNN–ViT	4.7	1.5	6.5
CNN–ViT–CBAM	**5.2**	**1.8**	**7.3**

## Data Availability

Data are unavailable due to privacy restrictions. No public involvement or specific reporting guidelines were applicable.
